# Linagliptin: farmacology, efficacy and safety in type 2 diabetes treatment

**DOI:** 10.1186/1758-5996-5-25

**Published:** 2013-05-22

**Authors:** Erika Paniago Guedes, Alexandre Hohl, Thais Gomes de Melo, Felipe Lauand

**Affiliations:** 1 Endocrinologist Board of Metabolism Outpatient Clinic from the 481 State Institute for Diabetes and Endocrinology (IEDE/RJ), Rio de Janeiro, RJ, Brazil; 2 Department of Endocrinology of Medical School of Graduate Studies, Pontifical Catholic University of Rio de Janeiro (PUC/RJ), Rio de Janeiro, RJ, Brazil; 3 Federal University of Santa Catarina (UFSC), Florianopolis, SC, Brazil; 4 Brazilian Society of Endocrinology and Metabolism - Santa Catarina state (SBEM-SC) - 2011/2012, Florianopolis, SC, Brazil; 5 Boehringer Ingelheim, São Paulo, SP, Brazil; 6 Eli Lilly, São Paulo, SP, Brazil; 7 Avenida das Américas, no. 2901, sala 305, Edifício Barra Business, Barra da Tijuca, Rio de Janeiro, RJ, 489 22631-030, Brazil

**Keywords:** DPP-4 inhibitor, Linagliptin, Efficacy, Safety, Renal impairment, Type 2 diabetes

## Abstract

Type 2 diabetes mellitus (T2DM) has a high prevalence and incidence around the world. The complex pathophysiology mechanism is among the barriers for diabetes treatment. Type 2 diabetes patients have dysfunction in incretin hormones (as glucagon-like peptide-1 or GLP-1, and glucose-dependent insulinotropic polypeptide or GIP). By inhibiting the dipeptidyl peptidase-4 (DPP-4) enzyme, it is possible to slow the inactivation of GLP-1 and GIP, promoting blood glucose level reduction in a glucose-dependent manner. Linagliptin is a highly specific and potent inhibitor of DPP-4 that is currently indicated for the treatment of type 2 diabetes. Clinical studies with linagliptin demonstrated efficacy in reducing glycated hemoglobin (HbA1c) levels in type 2 diabetes patients, while maintaining a placebo-like safety and tolerability profile. Linagliptin has an interesting pharmacokinetic profile in terms of its predominantly non-renal elimination and the main implication of this characteristic is that no dose adjustment is necessary in patients with renal disease. Also, no dose adjustment is required in patients with hepatic insufficiency, as well in elderly or obese patients. This article will review the pharmacokinetic profile, efficacy data and safety aspects of linagliptin in type 2 diabetes patients.

## Introduction

In recent decades, type 2 diabetes mellitus (T2DM) has reached epidemic proportions in all regions of the world, with increasing prevalence and incidence rates, in parallel to the obesity epidemic and the dissemination of occidental lifestyle [1]. It is estimated that in 2030, T2DM will achieve about 300 million people worldwide [2]. The micro- and macrovascular complications, associated with chronic hyperglycemia, represent a major public health problem. Cardiovascular diseases, blindness, renal failure and limb amputations are responsible for frequent hospitalizations and disabilities, resulting in high economic cost for patients and payers [1]. Early and intensive glycemic control is associated with reduction of these complications [3,4].

The pathophysiological defects involved in T2DM are numerous and complex. Insulin resistance (IR) is an event that precedes and predicts the hyperglycemia characteristic of T2DM, persisting throughout the course of the disease, and is therefore a therapeutic target in the whole evolution process. The liver, muscles, and fat tissue are directly involved in the IR mechanism [5]. The insulin deficit is the mechanism that promotes the increase of blood glucose levels, and at diagnosis of diabetes, the patient has already lost more than 80% of beta-cell function [5]. However, new mechanisms are reformulating the pathophysiological concept of T2DM, for example, the defect in the incretin system (Glucagon-like peptide-1 or GLP-1, and Gastric inhibitory polypeptide or GIP), promoting hyperglycemia by less stimulation of insulin secretion by pancreatic beta-cells, and less suppression of glucagon release by pancreatic alpha-cells [1]. GLP-1 and GIP are degraded by the dipeptidyl peptidase-4 (DPP-4) enzyme immediately after their secretion by intestinal L-cells [5]. DPP-4 inhibition is an important target for the T2DM treatment, providing increased GLP-1 concentration, with consequent increase of insulin secretion by pancreatic βcells and reduction of glucagon secretion from pancreatic α-cells, which in turn reduces hepatic glucose output [6].

The linagliptin is a DPP-4 inhibitor (or gliptin) with pharmacokinetic and pharmacodynamic characteristics that differenciate it from other inhibitors on the market [6]. In this article, aspects of the linagliptin pharmacology will be reviewed, in addition to safety, tolerability and efficacy data on monotherapy or combination therapy.

### Pharmacological aspects

#### Chemical structure

**Figure 1:**
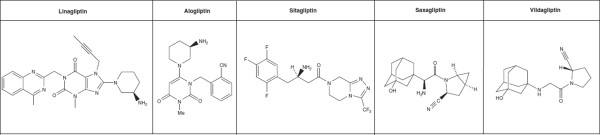
**Chemical structure of the DPP-4 inhibitors [**[9]**,**[10]**].**

The DPP-4 inhibitors can be divided, according to their chemical structure, into those that mimic the DPP-4 molecule (vildagliptin and saxagliptin) and those that do not (sitagliptin, alogliptin, and linagliptin). They inhibit the DPP-4 enzyme by competition, acting extra-cellularly [7]. The chemical structure of linagliptin (C_25_H_28_N_8_0_2_) has a xanthine base, differing from other drugs of the same class, and may reflect differences in pharmacokinetic and pharmacodynamic properties (Figure 1) [8,9]. The linagliptin also exhibits excellent selectivity for DPP-4 enzyme versus DPP-8 (40,000-fold) and DPP-9 (>10,000-fold), and a low selectivity for fibroblast activation protein-α (FAP-α) (Table 1) [6,7]. However, the DPP-8 and DPP-9 enzymes are intracellular and there is no evidence that less selective inhibitors, such as saxagliptin and vildagliptin, cross the cell membrane. Thus, despite questioning the possibility of an increased risk of adverse events with less selective inhibitors, the clinical relevance and significance of this selectivity are not established [6].

#### *Pharmacokinetic and pharmacodynamic data*

Linagliptin shows modest oral bioavailability, but is rapidly absorbed [9,10]. The maximum plasma concentration (C_max_) at steady state is reached on average 1.5 hours after administration of linagliptin 5 mg, once daily [11]. Linagliptin half-life (t_1/2_) is 131 hours [12]. No relevant food effects were observed on the absorption profile of linagliptin [10].

A strong binding of the inhibitor to the DPP-4 enzyme is important from the pharmacological point of view, to enable a 24-hour inhibition profile and the once-daily dosing. Linagliptin is a competitive, selective and reversible inhibitor, with ligand/receptor association (K_i_) of 1 nmol/L, indicating strong binding, and a low dissociation rate of the enzyme [8,9]. The maximal efficacy for in vitro DPP-4 inhibition is similar among all DPP-4 inhibitors, however linagliptin has greater potency (half maximal [50%] inhibitory concentration or IC_50_ = ~1 nM for linagliptin versus 19, 62, 50 and 24 nM for sitagliptin, vildagliptin, saxagliptin and alogliptin, respectively) [9]. In healthy male volunteers, linagliptin (2.5-600 mg) demonstrated dose-dependent inhibition of DPP-4 over 24 hours with a 5 mg dose inhibiting 86.1% of the enzyme activity [13]. It is important to note that the inhibition ≥ 80% of DPP-4 activity is the level which leads to the maximum glucose reduction [14].

**Table 1:** **In vitro selectivity of DPP-4 inhibitors (fold selectivity for DPP-4 versus other enzymes) (Adapted from Gupta et al.)**[8]

**Idpp4**	**FAP**_**α**_	**DPP-8**	**DPP-9**
Vildagliptin	285	270	32
Saxagliptin	?	390	77
Sitagliptin	>5.500	>2.660	>5.550
Alogliptin	>14.000	>14.000	>14.000
Linagliptin	89	40.000	>10.000

*FAP*_*α*_ Fibroblast activation protein _α_, *DPP-8* Dipeptidyl peptidase-8, *DPP-9* Dipeptidyl peptidase-9.

In plasma, most gliptins exhibit low binding rate to proteins [9]. Linagliptin, in turn, extensively binds to plasma proteins, and at the therapeutic dose of 5 mg, most of the drug is bound to proteins [9]. Among the drugs in this class, linagliptin, sitagliptin and alogliptin do not show appreciable metabolism in vivo; about 80% of the dose is eliminated unchanged [9]. In plasma, only the pharmacologically inactive metabolite CD18790 represents over 10% of the total drug concentration [15].

Unlike other DPP-4 inhibitors, linagliptin excretion is not performed by the kidneys, but rather through the enterohepatic system, unchanged [8]. This method of excretion may be in part because of the high plasma protein binding [9]. The implication of this mode of linagliptin excretion is that in patients with kidney disease, no dose adjustment is required [8].

### Clinical studies: efficacy assessment

#### *Monotherapy*

Although metformin is the first-line drug in the early pharmacological treatment of T2DM, some patients do not tolerate the drug, or exhibit contraindications [16]. In these cases, the DPP-4 inhibitors such as linagliptin can be an effective option for use as monotherapy. The efficacy of linagliptin as monotherapy, compared to placebo, was assessed in two studies of 12 and 24 weeks [17,18]. The linagliptin was significantly more effective than placebo in reducing glycated hemoglobin (HbA1c). Also independent of baseline HbA1c, the results were favorable for linagliptin; for baseline HbA1c ≥ 9.0%, 8.0% to < 9.0%, 7.5% to < 8.0% and <7.5%, the respective placebo-adjusted mean changes were −1.1% (p < 0.0001), -0.71% (p < 0.0001), -0.55% (p < 0.005) and −0.57 (p < 0.0001) [18].

The results of linagliptin monotherapy were also better than placebo in the secondary endpoints. There was more reduction in fasting plasma glucose (FPG) and 2-hour postprandial glucose (2hPPG) in the linagliptin group. The adjusted mean change in FPG was −1.3 mmol/L (p < 0.0001), and in 2hPPG was −3.2 mmol/L (p < 0.0001). The percentage of patients with HbA1c < 7% after 24 weeks was 25.2% (77/306) in the linagliptin group compared to 11.6% (17/147) in the placebo group (OR = 2.9, p = 0.0006) [18]. Besides, there was significant improvement in β-cell function markers (HOMA-%β, C-peptide, proinsulin-to-insulin ratio, and disposition index [DI]) in those receiving linagliptin [17,18].

Kawamori et al. also compared linagliptin monotherapy with voglibose, an α-glucosidase inhibitor, in a 26-week study [17]. More patients receiving linagliptin achieved HbA1c ≤ 7% (30.3%) when compared to voglibose (22.2%) [10]. The percentage of patients achieving a reduction ≥ 0.5% in HbA1c with linagliptin (57.2%) was also greater than those with voglibose (37.7%) (p < 0.0001) [10].

#### *Combination therapy*

•Combination with metformin

•Metformin is considered first-line therapy in most guidelines around the world. Following 3 years of T2DM diagnosis, approximately 50% of patients will require combination therapy [19]. Thus, the combination of metformin with another agent which complements its action will be performed in most type 2 diabetes patients. Graefe-Mody et al. evaluated in a randomized, open-label, crossover, single-center study, the potential pharmacokinetic and pharmacodynamic interaction between metformin and linagliptin. The coadministration of metformin 850 mg, three times daily and linagliptin (10 mg once daily) did not modify the pharmacological profile of each drug alone. This study suggested that the combination of metformin and linagliptin can be done safely in patients with T2DM, without requiring dose adjustment [20].

**Figure 2:**
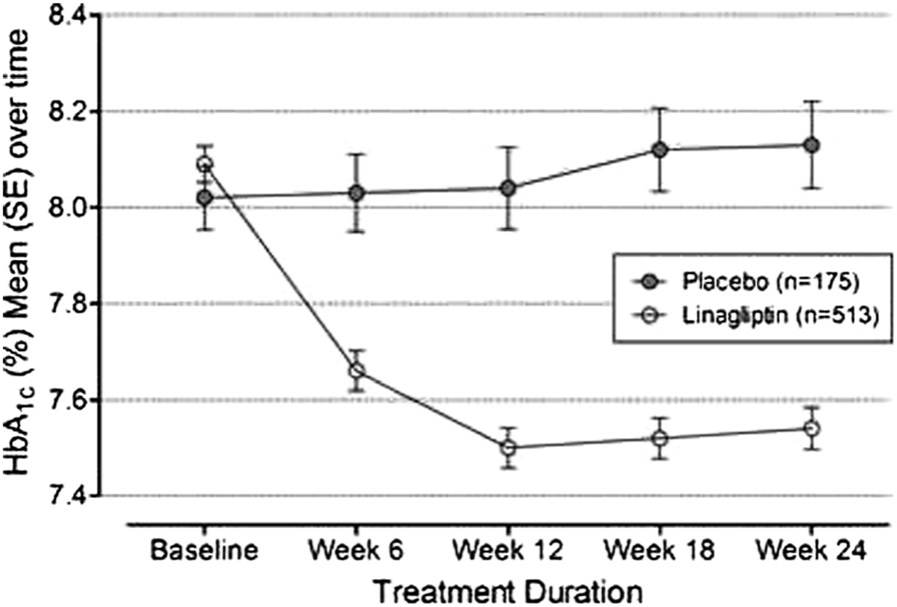
**Change over time in HbA1c, following treatment with linagliptin 5 mg or placebo for 24 weeks (reproduced from Taskinem et al.) [**[21]**].**

•In a 24-week study with about 700 patients, the addition of linagliptin to the therapeutic regimen in diabetic patients inadequately controlled on metformin, HbA1c reduction from baseline was 0.64% with the linagliptin versus placebo. The Figure 2 exhibits the HbA1c change over time [21]. The adjusted mean reduction in FPG was 1.2 mmol/L in the group with added linagliptin, and in 2hPPG was 3.7 mmol/L (p < 0.0001 for all comparisons) [22]. In another 12-week assessment, linagliptin 5 mg (single daily dose) was added to the metformin treatment (n = 333 patients), and was significantly more effective than placebo and the 1-mg or 10-mg doses [23].

•Haak et al. reported the findings of early combination of linagliptin and metformin in treatment-naïve diabetic patients, in a 24-week double-blind study [22]. Compared to metformin monotherapy (1000 mg), the early combination of metformin (1000 mg) and linagliptin (5 mg) was more effective in reducing HbA1c (−1.7% versus −0.8%, p <0.0001). Substantial reduction in FPG from baseline to Week 24 was found with the combination therapy [22].

•Combination with glitazone

•Linagliptin was also evaluated in combination with pioglitazone, in a 24-week investigation (n = 389 patients). The addition of linagliptin (5 mg) to pioglitazone (30 mg), both administered in a single daily dose, caused an adjusted mean placebo-corrected reduction of 0.5% in HbA1c levels from baseline at the end of 12 weeks, remaining constant until the 24th week. The group receiving the linagliptin/pioglitazone combination showed more significant reductions in FPG than placebo/pioglitazone group (p < 0.0001) [24]. More patients in the linagliptin/pioglitazone group (42.9%) achieved the target HbA1c < 7%, compared to the placebo-pioglitazone group (30.5%, p < 0.0051). The HbA1c reduction was greater in patients with baseline HbA1c ≥ 9% and treated with linagliptin in combination with pioglitazone (−1.49%). Body weight remained stable up to 24 weeks in the two groups. This combination can be interesting, even for early therapy in patients with an intolerance or contraindication to metformin.

•Combination with sulfonylurea

•In patients inadequately controlled on sulfonylurea alone, the addition of linagliptin 5 mg (single daily dose) proved more effective than the combination with placebo [25]. In this double-blind study, 245 patients were randomized to receive linagliptin (n = 161) or placebo (n = 84) for 18 weeks. The HbA1c reductions were significant in favor of linagliptin at Weeks 6, 12 and 18 (p < 0.0001) [26].

•Triple Combination

•The progressive failure of the pancreatic β-cell function contributes to evolutive character of the disease and the need for therapeutic adjustment. After 3 years of diagnosis, about 50% of diabetic patients will require combination therapy [19]. In a randomized, double-blind, placebo-controlled study, investigators have screened 1058 T2DM patients inadequately controlled on metformin (>1500 mg/day) and sulfonylurea (maximum tolerated dose) to receive the combination with linagliptin 5 mg (single daily dose) or placebo [27]. Assessing the total patients included, the adjusted mean change in HbA1c level was −0.72% in the linagliptin group compared with −0.10% in the placebo group, resulting in a difference of −0.62% (p < 0.0001) with placebo. Fewer patients receiving linagliptin required rescue therapy compared with placebo (5.4 versus 13.0%). More patients on linagliptin also achieved the target HbA1c. The importance of this study in practice is the possibility to improve glycemic control in patients already receiving two oral antidiabetic agents and who are outside the proposed targets [27].

### Comparison with sulfonylurea

The DPP-4 inhibitors promote glucose-dependent insulin secretion, i.e., in the presence of lower blood glucose values, the insulin release is not expected, which contributes to reduce the risk of hypoglycemia [16]. The sulfonylureas, in turn, are more associated with hypoglycemia since they do not exhibit glucose-dependent mechanism of action. In a 12-week analysis, 333 T2DM patients inadequately controlled on metformin monotherapy were randomized to receive linagliptin or glimepiride, in a single daily dose [25]. After 12 weeks, HbA1c has decreased both in the linagliptin or glimepiride groups. This study showed that linagliptin has the same efficacy as sulfonylureas, without risk of weight gain and hypoglycemia [25].

### Safety and tolerability

#### *Adverse events*

•Hypoglycemia

•DPP-4 inhibitors have a low risk of hypoglycemia due to their effect as glucose-dependent insulin secretagogue. The incidence of hypoglycaemia was 8.2% in patients receiving linagliptin and 5.1% in those receiving placebo. The somewhat higher incidence of hypoglycaemia associated with linagliptin was almost exclusively attributable to the combination with sulphonylurea. In studies where patients were receiving sulphonylurea, the incidence of hypoglycaemia was 20.7% and 13.3% in the linagliptin- and placebo-treated groups, respectively; notably, 38% of patients on sulphonylurea background therapy accounted for 96% of all hypoglycaemic events in the linagliptin-treated group [28]. As observed with other gliptins, the combination with linagliptin to patients inadequately controlled on metformin + sulfonylurea showed a higher occurrence of hypoglycemia than the placebo group (22.7% versus 14.8%) [27].

•Weight gain

•The T2DM treatment with glitazone, sulfonylurea, or insulin may be associated with weight gain. Most T2DM patients are overweight or obese, so it is not desirable to gain additional weight due to the treatment. DPP-4 inhibitors have a neutral effect on body weight [8]. Linagliptin showed no weight increase in monotherapy or in combination with metformin. In combination with pioglitazone, the linagliptin was associated with greater weight gain than placebo (2.3 kg versus 1.2 kg, p < 0.01), in a 24-week study, but these changes were minimal from baseline [24]. There was also no change in waist circumference with linagliptin treatment [24].

•Other adverse events

•The adverse events most frequently reported with DPP-4 inhibitors are mild infections (such as nasopharyngitis, urinary tract infection, and upper respiratory tract infections) and diarrhoea, back pain, headache and hypertension. Data presented with linagliptin indicate an overall incidence similar to placebo for these most frequently observed adverse events [28].

In 2008, the FDA (Food and Drug Administration) recommended the inclusion of a warning on the package insert of some drugs acting on the incretin system, after case reports of pancreatitis with the use of GLP-1 analogs [29]. Postmarketing analyses have also identified isolated cases of pancreatitis with DPP-4 inhibitors, but a cause-effect relationship was not identified. It is important to consider that patients with T2DM and hypertriglyceridemia exhibit increased risk of pancreatitis [7]. In clinical studies, 8 pancreatitis cases were reported in 4687 patients on linagliptin and no cases among 1183 patients receiving placebo; however, no relationship between linagliptin and pancreatitis has been established [13].

Despite the therapeutic dose used in practice being 5 mg, in a single daily dose, there seems to be no increased incidence of adverse events with increasing doses of linagliptin. Also, no changes in laboratory parameters or vital signs were observed in different studies with the drug. Moreover, the incidence of cutaneous and subcutaneous changes in linagliptin clinical trials was low when compared to placebo (0–1.3% versus 0–0.9%) [13].

### Patients with renal impairment

Linagliptin administration to patients with renal impairment does not require dosage adjustment [6]. When administered in a 5 mg dose, less than 1% of linagliptin is excreted unchanged in the urine. Thus, in therapeutic dose, renal excretion is a minor route of elimination for linagliptin, unlike other DPP-4 inhibitors [30]. When tested in patients with mild (creatinine clearance > 50 to ≥ 80 mL/min), moderate (creatinine clearance > 30 to ≤ 50 mL/min), and severe (creatinine clearance < 30 mL/min) renal impairment, in addition to the end-stage renal disease (creatinine clearance < 30 mL/min on hemodialysis), renal excretion of linagliptin remained unchanged and represents less than 7% in all groups [31]. In an analysis of 3 Phase III, randomized, placebo-controlled studies, the effect of renal function on the efficacy and safety of linagliptin was evaluated. Patients (n = 2141) were grouped according to renal function, and it was found that reductions in HbA1c with linagliptin did not differ among groups (mild, moderate, or severe renal impairment), as well as adverse event occurrences that were similar to placebo [31,32].

### Patients with liver failure

Linagliptin administration to patients with liver failure does not require dose adjustment. Although reductions occur in the linagliptin pharmacokinetic parameters as the liver failure degree increases, this has no impact on DPP-4 inhibition [6].

### Elderly patients

No changes were observed in safety and tolerability of linagliptin in patients aged over 65 years [6].

### Cardiovascular safety

The main cause of mortality in T2DM patients is cardiovascular disease (CVD) [33]. The gliptins may exert beneficial cardiovascular effects through different mechanisms (Table 2) [34]. Recent studies also demonstrated that intensive control may be associated with increased cardiovascular (CV) risk; therefore, another potential benefit of gliptins would be a low risk of hypoglycemia [35]. In studies using animal models, activation of GLP-1 receptor is associated with limiting the size of the area of myocardial infarction (MI) [36]. Furthermore, linagliptin has anti-oxidant properties, probably due to its xanthine-based molecular structure [37]. Even when administered in supratherapeutic doses, linagliptin does not prolong the QT interval [38].

A recent meta-analysis has assessed the cardiovascular safety profile of linagliptin in patients who participated in 8 Phase III studies. Of the 5239 patients, 3319 received linagliptin, and 1920 received a comparator (placebo, glimepiride, voglibose) [35]. A composite of CV death, stroke, MI, or hospitalization for unstable angina was considered as the primary endpoint. In the linagliptin group, the primary endpoint occurred in 11 (0.3%) patients, whereas in the comparator group there were 23 cases (1.2%), demonstrating lower risk in those who received linagliptin. An important conclusion is that, as other DPP-4 inhibitors have also demonstrated, linagliptin shows no increase in CV risk [35]. At present, various studies with DPP-4 inhibitors are currently ongoing to evaluate the safety and effects on cardiovascular endpoints (Table 3) [8].

### Drug interaction

In general, DPP-4 inhibitors have not demonstrated a significant activation or inhibition of CYP system enzymes, suggesting low potential for interaction with drugs metabolized by this pathway (except saxagliptin) [9]. Linagliptin is a substrate for CYP3A4/5, and CYP3A4 inhibition or induction by concomitant administration of other drugs would not cause significant change in exposure to linagliptin. Additionally, as linagliptin is just a weak competitive inhibitor of CYP3A4, there would be a less than 2-fold decrease in the clearance of other drugs metabolized by this pathway, so linagliptin is considered as having low potential for clinically relevant interactions [15].

**Table 2:** **Potential cardiovascular benefits of DPP-4 inhibitors**[38]

	
Neutral effect on body weight	Improved glycemic control, including PPG reduction
Decreased systolic blood pressure	Improved lipid profile
Reduction in C-reactive protein (CRP)	Improvement of endothelial dysfunction

**Table 3:** **DPP-4 inhibitors and cardiovascular risk: ongoing studies (adapted from**[8])

	
Sitagliptin	TECOS: Trial Evaluating Cardiovascular Outcomes with Sitagliptin
Saxagliptin	SAVOR: Saxagliptin Assessment of Vascular Outcomes Recorded in Patients with Diabetes Mellitus Trial
Alogliptin	EXAMINE: Examination of Cardiovascular Outcomes: Alogliptin vs Standard of Care in Patients with Type 2 Diabetes Mellitus and Acute Coronary Syndrome
Linagliptin	CAROLINA: Cardiovascular Outcome Study of Linagliptin Versus Glimepiride in Patients with Type 2 Diabetes

In different pharmacokinetic drug-drug interaction studies, linagliptin exhibited low potential for drug interaction [10]. Linagliptin did not change the pharmacokinetic steady state of ethinyl estradiol, levonorgestrel, digoxin, warfarin, glyburide, pioglitazone, simvastatin, and metformin [10]. Rifampicin, in turn, can reduce the exposure to linagliptin, suggesting that linagliptin efficacy may be reduced by concomitant use of these two drugs [12].

## Conclusions

Currently, DPP-4 inhibitors represent an important tool in the T2DM therapeutic setting, both for their efficacy in glycemic control, as well as for their safety profile. Regarding the effect on glycemic control, no significant differences have been evidenced among the gliptins. Linagliptin is a potent, long-acting, orally active DPP-4 inhibitor, with demonstrated efficacy as monotherapy and in combination therapy with other oral antidiabetic agents, when administered in a single daily dose. The primarily non-renal route of elimination of linagliptin differenciate it from other drugs in the same class; patients with kidney disease of any degree do not require dose reduction of linagliptin. The risk of adverse events with linagliptin monotherapy is similar to placebo. Its effect on body weight is considered neutral and the hypoglycemia occurrence is very low. Therefore, linagliptin can contribute to the achievement of glycemic targets, with safety and tolerability, even in special difficult-to-manage situations, as in those patients with renal impairment.

## Abbreviations

T2DM: Type 2 diabetes mellitus; GLP-1: Glucagon-like peptide-1; GIP: Glucose-dependent insulinotropic polypeptide; DPP-4: Dipeptidyl peptidase-4; HbA1c: Glycated hemoglobin; Cmax: Maximum plasma concentration; t1/2: Half-life; IC50: Half maximal (50%) inhibitory concentration; FPG: Fasting plasma glucose; 2hPPG: 2-hour postprandial glucose; FDA: Food and drug administration; CVD: Cardiovascular disease; CV: Cardiovascular; MI: Myocardial Infarction

## Competing interests

Guedes EP has received lecture and/or consultancy fees from companies with interest in type 2 diabetes therapies (Astra Zeneca, Bristol-Meyers-Squibb, Novo Nordisk, Boehringer-Ingelheim, Torrent and Abbott).

Hohl A has received lecture and/or consultancy fees from companies with interest in type 2 diabetes therapies (Merck Sharp Dohme, Bristol-Meyers-Squibb, Novartis, Merck Serono, Boehringer-Ingelheim, Eli Lilly, Sanofi and Abbott).

Melo TG is medical manager for Boehringer-Ingelheim do Brasil.

Lauand F is medical manager for Eli Lilly do Brasil.

## Authors’ contributions

All authors participated equally in the development of this paper. All authors also read and approved the final manuscript.
